# Oyster Peptide-Zinc Complex Ameliorates Di-(2-ethylhexyl) Phthalate-Induced Testis Injury in Male Mice and Improving Gut Microbiota

**DOI:** 10.3390/foods13010093

**Published:** 2023-12-27

**Authors:** Zhen Lu, Qianqian Huang, Fujia Chen, Enzhong Li, Haisheng Lin, Xiaoming Qin

**Affiliations:** 1 Guangdong Provincial Key Laboratory of Aquatic Products Processing and Safety, Guangdong Provincial Science and Technology Innovation Center for Subtropical Fruit and Vegetable Processing, College of Food Science and Technology, Guangdong Ocean University, Zhanjiang 524088, China; 1122103003@stu.gdou.edu.cn (Z.L.);; 2 School of Biological and Food Processing Engineering, Huanghuai University, Zhumadian 463000, China; 3 National Research and Development Branch Center for Shellfish Processing, Zhanjiang 524088, China

**Keywords:** oyster peptide-zinc complex, DEHP, testis injury, zinc, intestinal microbiota

## Abstract

Di-(2-ethylhexyl) phthalate (DEHP) is a widely used plasticizer, which can cause damage to male reproductive organs, especially the atrophy of the testis. Meanwhile, DEHP can also lead to a decrease in testicular zinc content, but the role of zinc remains unclear. This study aims to prepare oyster peptide-zinc complex (OPZC) to alleviate DEHP-induced reproductive damage in mice. OPZC was successfully obtained through electron microscopy, X-ray diffraction, and thermogravimetric analysis, with stable structure and high water-solubility. Low dose oyster peptide-zinc complex (OPZCL) significantly reduced the reproductive damage caused by DEHP in mice. Further research had shown that OPZCL restored the content of serum hormones and the activity of oxidative stress kinases to normal, while also normalizing testicular zinc and selenium levels. In addition, it also recovered the disorder of gut microbiota, reduced the proportion of *Bacteroides*, increased the abundance of *Ligilactobacillus*, and restored the proportion of *Acidobacteriota, Chloroflexi*, and *Proteobacteria*. Therefore, OPZCL can relieve the reproductive damage caused by DEHP in mice by restoring testicular zinc homeostasis and the composition of intestinal microbiota, indicating that OPZCL has a potential protective effect on male reproductive health.

## 1. Introduction

Di-(2-ethylhexyl) phthalate (DEHP) is often used as plasticizer in the plastics industry to produce plastic products [1]. It can be found in daily necessities, including food packaging, cosmetics, and plastic containers. However, DEHP can easily leach out of the daily necessities, and finally be absorbed by the human body, which can lead to reproductive toxicity [2,3]. It is reported that DEHP may cause oxidative stress in male reproductive organs, such as the testis and epididymis, leading to impaired spermatogenesis and altered the release of reproductive hormones [2,4]. Exposure to DEHP during pregnancy has been linked to testicular reproductive damage in F1-F3 generation mice [5]. Various studies have confirmed that exposure to phthalates can lead to complex interactions between gut microbiota imbalance and host pathophysiology. Recently, the study shows that DEHP causes damage to the gut microbiota, which can also regulate the endocrine function of the testes [6,7]. Yu et al. had found out that the long-term exposure to low-dose DEHP in rats could lead to the dysregulation of gut microbiota [8].

There are concerns about the growing exposure to DEHP contaminants due to the increasing consumption of packaged and fast foods by youth, as well as the frequent consumption of take-out products by college students [9,10]. The continuous exposure to endocrine disruptors is believed to be part of the reason for the decline in the human fertility rate over the past 50 years [11]. Foster et al. had found that the zinc content in the testes of rats was lost after being treated with phthalates (DEHP), it was believed to be an inducing factor of testicular atrophy [12,13]. Zinc is closely associated with various aspects of spermatogenesis, and the zinc content is very high in the adult testes [14,15]. People ingest some food matrices with components such as phytic acid, which tends to form insoluble complexes with zinc ions, affecting the digestion and absorption of metallic elements [16]. Current research has found that peptide–zinc chelates have better stability, and animal studies have shown that peptide–zinc chelates have better bioavailability results than zinc gluconate and zinc sulfate [17,18]. Therefore, mineral chelate complexes are considered to be promising fortification agents that can be protected from inhibitors until they are absorbed by the intestine, avoiding any unfavorable changes [19].

Oysters are rich in protein and widely distributed marine biological resources worldwide. Oysters were approved by the Ministry of Health of China as dual-use materials for medicine and food [20]. In previous studies, oyster peptides were proven to have a positive effect on male reproduction function, including higher antioxidant activity and protective effects against reproductive impairment [21,22,23]. However, the protective effects of oyster peptide–zinc complex (OPZC) on reproductive impairment have not been systematically reported. So, OPZC have the potential to protect reproductive function against DEHP damage and regulate the gut microbiota. This study aimed to load OPZC to promote the absorption of zinc and protect against testicular injury in DEHP-treated mice.

## 2. Materials and Methods

### 2.1. Materials

Crude oyster peptides (molecular weight is between 180 Da and 1000 Da) were provided by Hainan Shengmeinuo Biotechnology Co., Ltd. (Wenchang, China). All chemicals and reagents used were of analytical grade and commercially available (Shanghai, China).

### 2.2. Preparation of Ethanol-Soluble Oyster Peptide and Oyster Peptide–Zinc Complex

After alcohol extraction, crude oyster peptide was centrifuged at 15,500× *g* for 30 min at 4 °C. The supernatant was concentrated and freeze-dried to obtain ethanol soluble oyster peptide (OP) [24].

The oyster peptide-zinc complex (OPZC) was prepared according to the method of Chen et al. with some modifications [25]. The mixture reacts in a water bath at 40 °C for an hour (the mass ratio of OP to zinc sulfate is 1:3, pH 6.5), then use ice cubes to cool down. After being centrifuged at 4 °C 9900× *g* for 20 min, and the supernatant was collected. Subsequently, add anhydrous ethanol in a 1:3 (supernatant: anhydrous ethanol) volume ratio and react for 5 h. After centrifugation at 9900× *g* for 10 min, it was washed several times with 80% ethanol to remove free zinc ions, and then the OPZC was finally obtained [26]. The freeze-dried OPZC powder was stored at −20 °C. The zinc content of OPZC was measured by Agilent 7900 ICP-MS (Santa Clara, CA, USA) (zinc content: 95.61 mg/g).

### 2.3. Amino Acid Composition Analysis

The OP or the OPZC was filtered, deacidified, and dissolved for amino acid analysis. An amino acid analyzer (L-8900, Hitachi, Tokyo, Japan) was used to determine the amino acid composition of OP and OPZC.

### 2.4. Mass Spectrometry Identification of OPZC and Molecular Docking Simulations

The OPZC chelate peptides sequence was analyzed using Q Exactive™ HF-X Hybrid Quadrupole-Orbitrap™ Mass Spectrometer (Thermo Fisher Scientific, Waltham, MA, USA).

In this experiment, the homology modeling of peptide sequences was performed by Rosetta 3.14 [27], and the modeling template was derived from the higher ranked structures in blast results. The zinc ion structure was obtained using pubchem, and the peptide and zinc ion were docked by Autodock vina 1.1.2 [28]. Meanwhile, the peptide was pocketed, and the pocket was set to be a cubic box covering the peptide with the size of 5 Å × 5 Å × 5 Å. The docking results were analyzed using Pymol 2.5 (https://pymol.org/2/, accessed on 20 October 2023).

### 2.5. Analysis of Physicochemical Characteristics

OP and OPZC were evaluated by scanning electron microscopy (SEM, DSM 940A, ZEISS, Jena, Germany), X-ray microanalysis by energy dispersion (EDX), and X-ray diffraction diffractogram (XRD) thermogravimetry (TG) (Netzsch STA 2500, Selb, Germany).

### 2.6. Animals and Experimental Design

All animal experimental protocols and procedures in this study were approved by the Experimental Animal Committee of Guangdong Ocean University (GDOU-LAE-2022-035). All male ICR mice (3 weeks old, 15–17 g) (Animal license number SCXK (Beijing, China) 2019-0010) were purchased from Guangzhou Yan cheng Biotechnology Co., Ltd. (Beijing, China) and housed in a standard animal husbandry room. After 1 week recovery, the surviving mice were randomly divided into seven groups (n = 8) and treated orally for six weeks included: group A (control) received corn oil; group B (DEHP) received 1000 mg/kg bw/day DEHP (TCI, Tokyo, Japan) in corn oil; group C (DEHP.ZnSO_4_) treated with ZnSO_4_ (zinc content: 8.14 mg/kg) plus DEHP; group D (DEHP.OP) treated with OP (110 mg/kg) plus DEHP; group E (DEHP.OPZCL) treated with OPZC (zinc content: 5 mg/kg) plus DEHP; group F (DEHP.OPZCM) treated with OPZC (zinc content: 15 mg/kg) plus DEHP; and group G (DEHP.OPZCH) treated with OPZC (zinc content: 25 mg/kg) plus DEHP.

### 2.7. Anogenital Distance, Body, and Organ Weight

Animals were executed at the same age of 42 days. Anogenital distance (AGD) and body weight were measured, followed by the dissection and weighing of testes, seminal vesicles, liver, and kidney organs. These organs were stored at −80 °C for further analysis, the right testis was used for histologic analysis.

### 2.8. Sperm Morphology and Viability

The entire right epididymis was placed in 1 mL of preheated saline and cut into small segments. After incubation in a 37 °C environment, the sperm suspension was dropped into a Neobar’s hemocytometer and the sperm count was estimated under a coverslip. The rate of abnormal sperm morphology was assessed and calculated by observing the sperm staining images using the Quick sperm stain kit (Nanjing Jiancheng Bioengineering Institute, Nanjing, China). Sperm parameters were analyzed according to the method adopted by Qiu et al. [29].

### 2.9. Testis Histopathological and TUNEL Apoptosis Assay

Fix the right testicle in 4% paraformaldehyde and embed it in paraffin. Stain the tissue slice with hematoxylin eosin. Finally, the tissue morphology and state of spermatogenesis was observed under an optical microscope (Nikon Corporation, Tokyo, Japan). Take the testicular tissue from each group of mice and detect the apoptosis of spermatogenic cells in testicular tissue using the TUNEL apoptosis assay kit. Red fluorescent cells are considered apoptotic cells.

### 2.10. Measurements of Enzyme

The levels of total superoxide dismutase (T-SOD), copper–zinc superoxide dismutase (CuZn-SOD), malondialdehyde (MDA), and lactate dehydrogenase (LDH) were measured in testicular tissues according to the kit description (Nanjing Jianjian Bioengineering Institute, Nanjing, China).

### 2.11. Determination of Serum Hormone Levels, Zinc Concentration, and MT Concentration

The serum levels of sex hormones such as testosterone (T), estradiol (E2), follicle-stimulating hormone (FSH), and luteinizing hormone (LH), as well as the levels of zinc ions and metallothionein MT were all measured by enzyme-linked immunosorbent assay kits in mice (Mmbio, Yancheng, China).

### 2.12. Minerals Concentrations Determination

Trace and mineral elements were measured by Agilent 7500CE ICP-MS and Agilent 720ES ICP-OES (Agilent Technologies Inc., Tokyo, Japan) in testis samples. The content of Mg, Zn, and Fe is relatively high, so ICP-OES was chosen for testing. The contents of Cu, Mn, and Se is relatively low, so ICP-MS was chosen for testing.

### 2.13. Gut Microbiota Analysis

Fresh fecal samples were collected from the ICR mice in DEHP-induced model at week 6 and stored at −80 °C. For bacterial diversity analysis, the V3–V4 variable region of the 16 S rRNA genes was amplified using the universal primers 338 F and 806 R [30].

### 2.14. Statistical Analysis

Results were expressed as the mean ± standard deviation (SD) and analyzed by SPSS version 26 (IBM Corp., Armonk, NY, USA). All the experimental data were analyzed using one-way ANOVA. Differences at *p* < 0.05 were considered statistically significant.

## 3. Results

### 3.1. Amino Acid Composition of OP and OPZC

The amino acid content and composition of OP and OPZC are shown in Table 1. The amino acid composition and content of OPZC and OP had undergone significant changes. The total amino acid content of OPZC (41.35 ± 0.76%) had decreased by 6.85% compared to OP (48.20 ± 0.07%), which was attributed to the presence of zinc in OPZC. In addition, this study also found that the relative content of aspartic acid (Asp), cysteine (Cys), histidine (His), lysine (Lys), and arginine (Arg) significantly increased (*p* < 0.05), indicating that these amino acids play an important role in zinc chelation [31].

### 3.2. Main Peptide Sequences of OPZC and Molecular Docking Result

The peptide sequence of OPZC was determined by LC-MS/MS, and the molecular weight of OPZC peptide was between 600 and 2000 Da, confirming that low-molecular-weight peptides have a better metal chelating activity. As shown in Table 2, 16 identified peptide amino acid sequences with high scores and abundance were selected. The bioactivity probability ranged from 4.73% to 82.23% was evaluated using PeptideRanker bioinformatics tool (http://distilldeep.ucd.ie/PeptideRanker/ (accessed on 8 October 2023).

**Figure 1:**
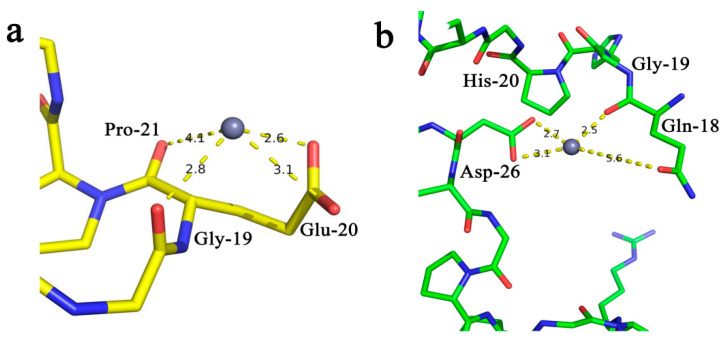
Molecular docking of zinc-chelating peptides: (**a**) Interaction between Gly-19, Glu-20, and Pro-21 in peptide GEPGPEGPAGPIGPR with zinc; (**b**) Interaction between Gln-18, Gly-19, His-20, and Asp26 in peptide GHPGLPGDAGPEGPR with zinc. Note: the Grey ball sphere represents zinc.

Molecular docking is widely used to predict the interaction between ligands and receptors. GEPGPEGPAGPIGPR and GHPGLPGDAGPEGPR were chosen for molecular docking due to their predicted high biological activity. The docking results showed that the type of interaction between the zinc ion and carboxyl group was the charge type. The chelation position, and the distance of OPZC peptide-to-zinc ion were showed in Figure 1a,b. Molecular docking analysis indicates that glutamine (Gln), glutamate (Glu), and histidine (His), aspartate (Asp) may play an important role in the chelating ability of zinc.

### 3.3. Physicochemical Characteristics Result of the OP and OPZC

**Figure 2:**
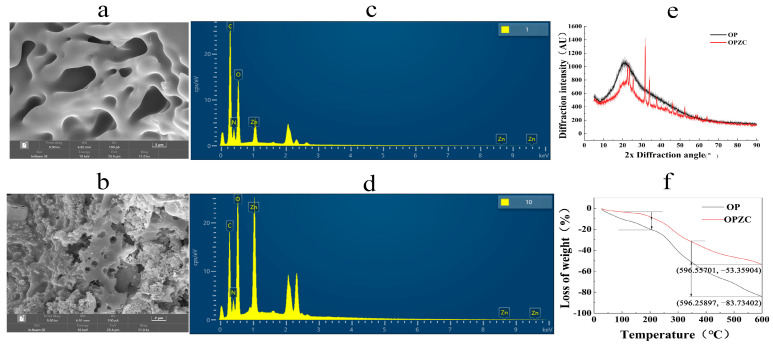
The physical and chemical characterization of OP and OPZC: (**a**,**b**) SEM images (×5.0 kx); (**c**,**d**) energy spectrum; (**e**) X-ray diffraction pattern; and (**f**) thermogravimetric curves.

The surface of OP (Figure 2a) was porous, smooth, and relatively loose; however, the OPZC (Figure 2b) exhibited rough spherical particle aggregates, mainly due to the combination of peptides and zinc ions through ion bonds and coordination bonds. The differences in the morphological features indicated that the interactions between the zinc and functional groups strongly determine the formation of nanostructures [32]. The surface elemental composition of OP and OPZC were analyzed by SEM-EDX. The results showed that OP (Figure 2c) was mainly composed of four elements, C (53.72%), N (16.13%), O (26.0%), and Zn (4.15%), while after chelation, the surface elemental composition of OPZC (Figure 2d) had changed to C (38.77%), N (11.56%), O (30.02%), and Zn (19.65%). It was confirmed that the oyster peptide undergoes a chemical reaction with zinc ions to generate zinc chelating peptides.

OP had a wide dispersion peak near 20°, presenting an amorphous state (Figure 2e). After chelating with zinc, many new diffraction peaks appeared at 22°, 25°, 32°, 34°, 37°, 46°, and 52°. The emergence of new peaks were the result of the formation of chelates between OP and zinc ions. The stability of OPZC was attributed to the eutectic structure, and had a profound influence on its anti-hydrolysis ability.

As shown in Figure 2f, the temperature for decomposing OPZC was significantly higher than OP. At 600 °C, the weight loss of OP was 83.73%; however, the weight loss of OPZC was 53.40%, indicating that the OPZC had formed stable chemical bonds which need more energy to decompose.

### 3.4. Effects of OPZC on Organ Coefficient and Anogenital Distance of DEHP-Induced Mice

**Figure 3:**
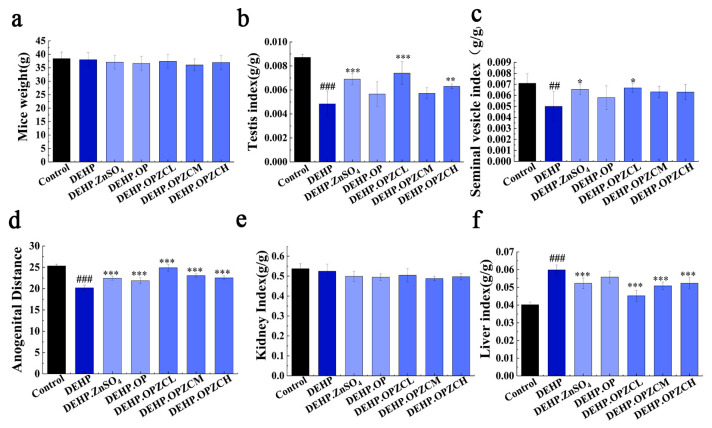
Effect of OPZC on final body weight (**a**); testis index (**b**); seminal vesicle index (**c**); anogenital distance (**d**); kidney index (**e**); and liver index (**f**) of the ICR mice treated with DEHP. The data are expressed as mean ± SD. Compared with the Control group, ## means very significant difference (*p* < 0.01), and ### means extremely significant difference (*p* < 0.001). Compared with the DEHP group, * means a significant difference (*p* < 0.05), ** means very significant difference (*p* < 0.01), and *** means extremely significant difference (*p* < 0.001).

The organ coefficient of the testis is a commonly used detection indicator in animal toxicology experiments. As shown in the figure (Figure 3), the results revealed that the final body weight (Figure 3a) and kidney index (Figure 3e) had no significant change. Changes in testicular tissue structure can seriously affect the quality and quantity of sperm. The testis index and seminal vesicle index in different treatment groups was presented in Figure 3b,c and showed that DEHP exposure decreased both of them (*p* < 0.01), while the treatment of ZnSO_4_ and OPZCL ameliorated the testicular weight loss compared with the DEHP group (*p* < 0.05). Moreover, Figure 3d showed that the DEHP induced a short anogenital distance which were reversed by treatment of ZnSO_4_ and OPZC. Furthermore, DEHP had a significant impact on the liver index (Figure 3f), and the zinc sulfate and OPZC groups were able to alleviate the increase in liver index.

### 3.5. Effects of OPZC on Sperm Morphology and Viability of DEHP-Induced Mice

**Figure 4:**
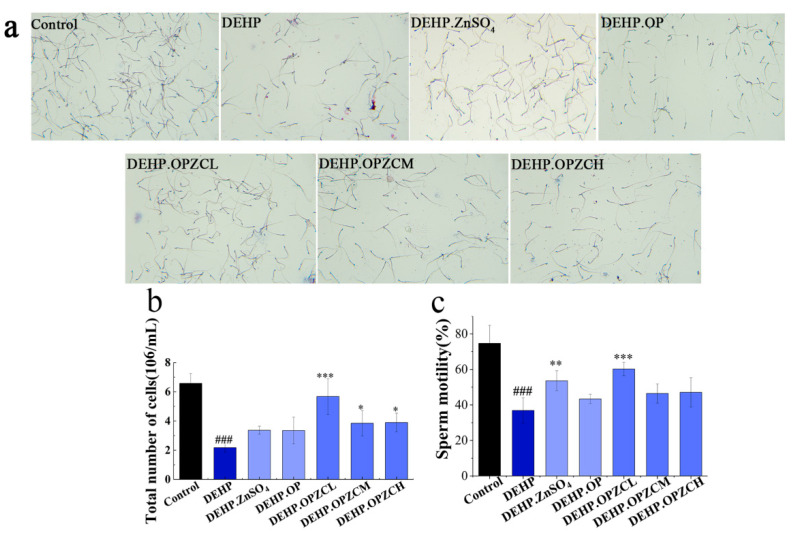
Effect of OPZC on sperm quality of ICR mice treated with DEHP. (**a**) Sperm morphology (200×); (**b**) total number of the sperm; (**c**) sperm motility. The data are expressed as mean ± SD. Compared with the control group, ### means an extremely significant difference (*p* < 0.001). Compared with the DEHP group, * means a significant difference (*p* < 0.05), ** means very significant difference (*p* < 0.01), and *** means an extremely significant difference (*p* < 0.001).

After DEHP treatment, the abnormal morphology of mouse sperm significantly increased, such as acrosome abnormalities, chubby head, curved neck, short tail, and curled tail. After the OPZC intervention, the sperm morphology returned to normal (Figure 4a). DEHP significantly reduced the number and motility of mouse sperm, and increased the rate of sperm deformity, OPZCL, OPZCM, OPZCH, and zinc sulfate increased the number and motility of mouse sperm, and significantly reduced the rate of sperm deformity. Among them, low dose oyster peptide-zinc complex (OPZCL) had the best effect (Figure 4b,c). In this research, ZnSO_4_ and OP was chosen as the positive control. As shown in Figure 4, ZnSO_4_ treatment significantly ameliorated the sperm damage induced by DEHP (*p* < 0.01), while the OP group had a poor treatment outcome.

### 3.6. Histopathological and TUNEL Apoptosis Assay Analysis

**Figure 5:**
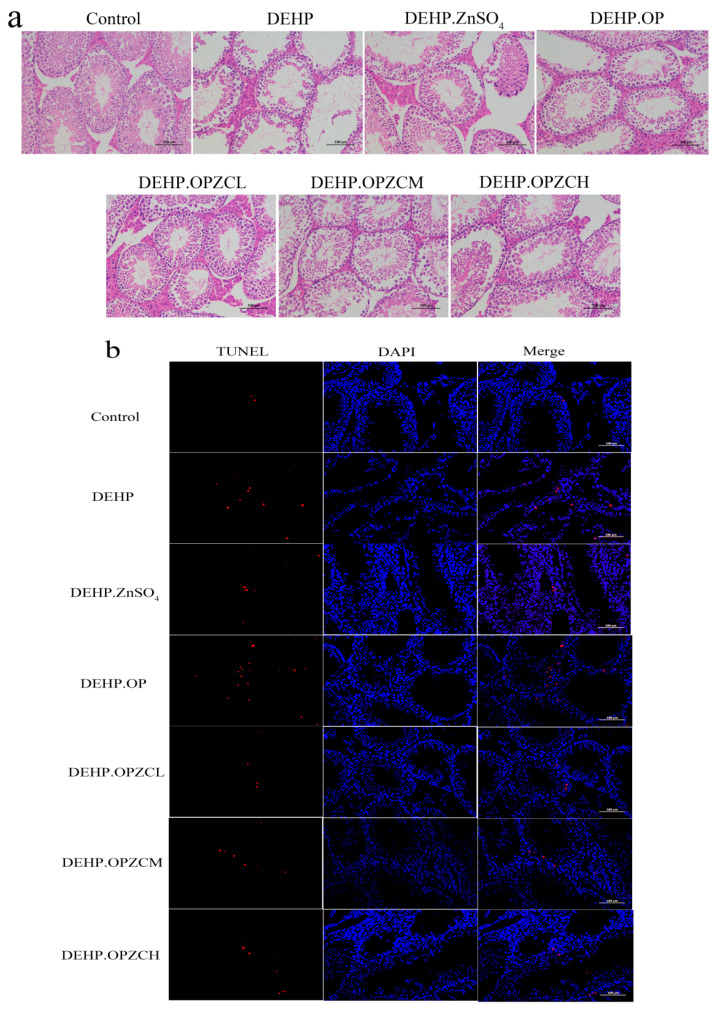

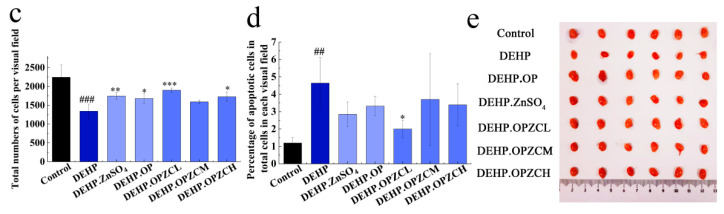
Effects of OPZC on the testicular injury of mice induced by DEHP: (**a**) Histopathology with HE staining (200×); (**b**) TUNEL assay (apoptotic cells:red fluorescence); (**c**) Total number of cells in each group; (**d**) Effects of OPZC on apoptotic index; and (**e**) Testis morphology. The data are expressed as mean ± SD. Compared with the Control group, ## means very significant difference (*p* < 0.01), and ### means extremely significant difference (*p* < 0.001). Compared with the DEHP group, * means a significant difference (*p* < 0.05), ** means very significant difference (*p* < 0.01), and *** means extremely significant difference (*p* < 0.001).

Histological analysis on the tissue sections of H&E staining showed that OPZC treatment protected testis against the damage caused by DEHP (Figure 5a). In the control group, the seminiferous tubules were well developed with a regular cell arrangement. However, the seminiferous tubules in the DEHP group were markedly distorted with wide lumina and no spermatozoa. In this research, ZnSO_4_ treatment provided a significant prevention of DEHP induced testicular toxicity, but the OP treatment effect was not good. Moreover, OPZCL, OPZCM, and OPZCH showed an obvious prevention of DEHP-induced testicular toxicity, especially OPZCL, which had the best effect. The structure of the spermatogenic tubules in the OPZCL group returned to normal, with the mild loss and normal arrangement of spermatogenic cells.

The TUNEL staining results showed that compared with the control group, there was a significant increase in positive apoptotic cells (red fluorescent cells) in the DEHP group, and there were also more positive cells in the OP and OPZCM treatment groups (Figure 5b). The number of red fluorescent cells in the OPZCH, ZnSO_4_, and OPZCL groups decreased, and ZnSO_4_ and OPZCL groups returned to a better state. The quantitative statistical analysis of cell numbers in TUNEL-stained tissue sections showed that the total number of cells in the testicular tissues of mice in the DEHP model group was significantly reduced (Figure 5c). And the proportion of apoptotic cells was significantly increased compared with the control group (Figure 5d). OPZCL and zinc sulfate significantly ameliorated the DEHP-induced reduction in the total number of cells in testicular tissues, and this result also confirmed the histopathological detection results of HE staining. Figure 5e showed the morphology of the testes, as the testis of mice became smaller and showed atrophy after DEHP treatment, and the zinc sulphate group and OPZC group were able to restore the normal size of testes.

### 3.7. OPZC Increased Testicular Marker Enzyme Activity and Reduced Oxidative Stress Induced by DEHP

**Figure 6:**
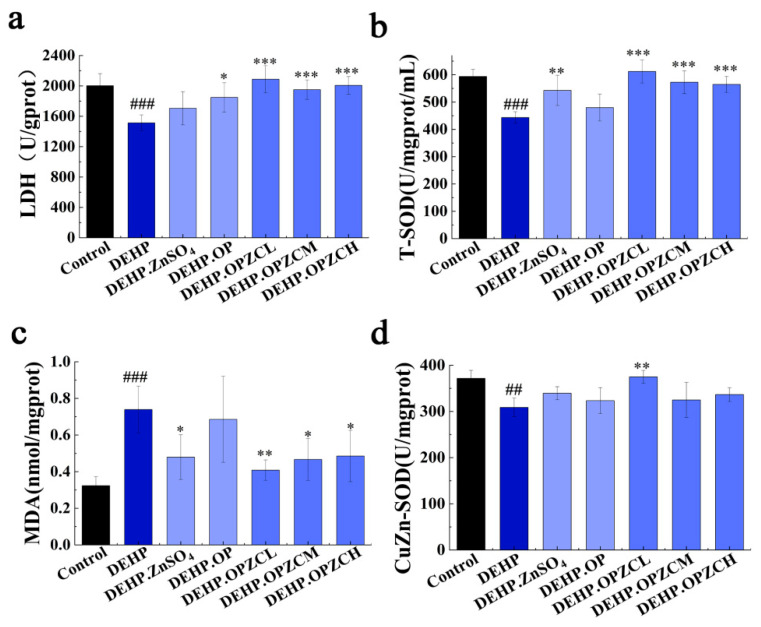
Effects of OPZC on testicular marker enzymes and the biomarkers of oxidative stress in the testes tissues of mice treated with DEHP: (**a**) Lactate dehydrogenase (LDH) level; (**b**) Total superoxide dismutase (T-SOD) level; (**c**) Malondialdehyde (MDA) level; and (**d**) Copper (Cu)–Zn superoxide dismutase (CuZn-SOD) level. The data are expressed as mean ± SD. The data are expressed as mean ± SD. Compared with the Control group, ## means very significant difference (*p* < 0.01), and ### means extremely significant difference (*p* < 0.001). Compared with the DEHP group, * means a significant difference (*p* < 0.05), ** means very significant difference (*p* < 0.01), and *** means extremely significant difference (*p* < 0.001).

The levels of testis oxidation and antioxidant indicators are shown in Figure 6. DEHP significantly decreased the level of LDH enzymes in testis tissue (*p* < 0.01) (Figure 6a), and OPZC supplementation resulted in a more significant amelioration in LDH production in mice treated with DEHP (*p* < 0.01). Oxidative stress is one of the main pathogenic mechanisms of DEHP-induced testicular tissue damage. DEHP destroyed the antioxidant system of the testicles, including the total superoxide dismutase (T-SOD) level (Figure 6b) and copper (Cu)–Zn superoxide dismutase (CuZn-SOD) level (Figure 6d) and raised MDA (Figure 6c). As expected, the OPZC treatment increased the activity of T-SOD and CuZn-SOD, and reduced the level of MDA (*p* < 0.001). These results suggest that OPZC can restore the redox status by increasing the activity of endogenous antioxidant enzymes (LDH, T-SOD, CuZn-SOD) and decreasing malondialdehyde (MDA).

### 3.8. Effects of OPZC on Serum Sex Hormones, Zinc Concentration, and MT Concentration

**Figure 7:**
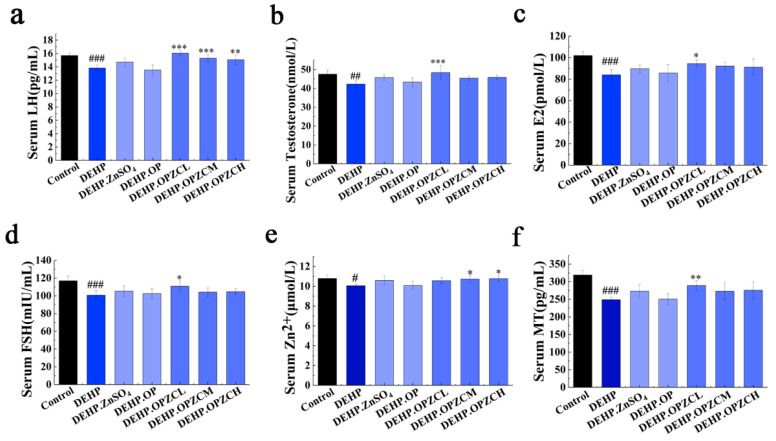
Effects of OPZC on serum reproductive hormone level, zinc, and MT concentration of mice treated with DEHP. (**a**) Luteinizing hormone (LH); (**b**) Testosterone (T); (**c**) Estradiol (E2); (**d**) Follicle-stimulating hormone (FSH); (**e**) Zinc concentration; and (**f**) Metallothionein (MT). The data are expressed as mean ± SD. The data are expressed as mean ± SD. Compared with the Control group, # means a significant difference (*p* < 0.05), ## means very significant difference (*p* < 0.01), and ### means extremely significant difference (*p* < 0.001). Compared with the DEHP group, * means a significant difference (*p* < 0.05), ** means very significant difference (*p* < 0.01), and *** means extremely significant difference (*p* < 0.001).

Relative hormone levels in males can reflect male fertility to some extent, so we compared the hormone levels in mice from different treatment groups. The concentration of luteinizing hormone (LH) (Figure 7a), serum testosterone (T) (Figure 7b), estradiol (E2) (Figure 7c), and follicle stimulating hormone (FSH) (Figure 5d) were significantly decreased after exposure to DEHP compared with the control group (*p* < 0.05). The OPZCL treatment restored serum testosterone, estradiol, follicle-stimulating hormone (FSH), and luteinizing hormone (LH) levels in mice close to those of the control group compared to the DEHP group (*p* < 0.05).

Several zinc-dependent proteins and enzymes are frequently used as biomarkers to measure zinc levels in organisms, such as metallothionein (MT). The average serum zinc level (Figure 7e) and MT (Figure 7f) in the DEHP group was significantly lower than the control group (*p* < 0.05). Moreover, the serum zinc level in the OPZCM and OPZCH group significantly increased compared to those in the DEHP group, while the OPZCL group significantly increased the serum MT (*p* < 0.01). These results suggest that OPZC supplementation can raise serum zinc and MT after DEHP injury.

### 3.9. Effect of OPZC on the Content of Various Mineral Elements in Testicular Tissues of DEHP-Treated Mice

A previous study found that DEHP may alter the balance of trace element and mineral concentrations in serum, which will damage the cellular and biochemical processes in organisms [33].

**Figure 8:**
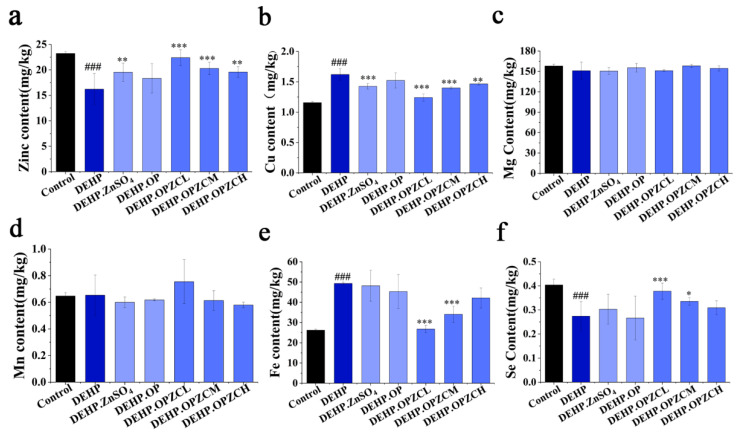
Effects of OPZC on the mineral concentration in the testis of DEHP-treated mice. (**a**) Zn; (**b**) Cu; (**c**) Mg; (**d**) Mn; (**e**) Fe; and (**f**) Se. The data are expressed as mean ± SD. The data are expressed as mean ± SD. Compared with the Control group, ### means extremely significant difference (*p* < 0.001). Compared with the DEHP group, * means a significant difference (*p* < 0.05), ** means very significant difference (*p* < 0.01), and *** means extremely significant difference (*p* < 0.001).

Concentrations of various trace elements including Zn, Cu, Mg, Mn, Fe, and Se were investigated in the DEHP administered testes samples. According to our data, exposure to DEHP decreased the concentrations of zinc (Zn) (Figure 8a) and selenium (Se) (Figure 8f) in testis, and OPZCL significantly improved the homeostasis of these two elements within the testis tissues (*p* < 0.01). Compared with the control, the DEHP model group increased the concentrations of copper (Cu) (Figure 8b) and iron (Fe) (Figure 8e) in the testis, and OPZCL and OPZCM effectively improved the unstable state (*p* < 0.001). However, no significant change was observed in the concentrations Mg (Figure 8c) and Mn (Figure 8d) elements compared to the control group.

### 3.10. Analysis of Gut Microbiota after OPZC Treatment

**Figure 9:**
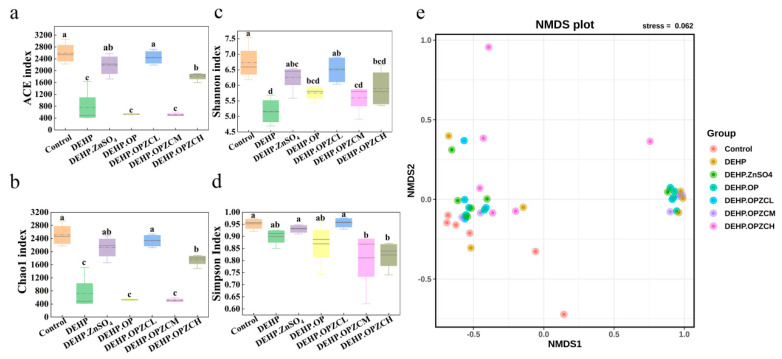
OPZC regulates gut microbiota of mice treated with DEHP. (**a**) ACE; (**b**) Chao1; (**c**) Shannon; (**d**) Simpson; and (**e**) NMDS analysis. Note: Different lowercase letters represent significant differences between different groups. Different superscripts letters are statistically significant differences (*p* < 0.05).

**Figure 10:**
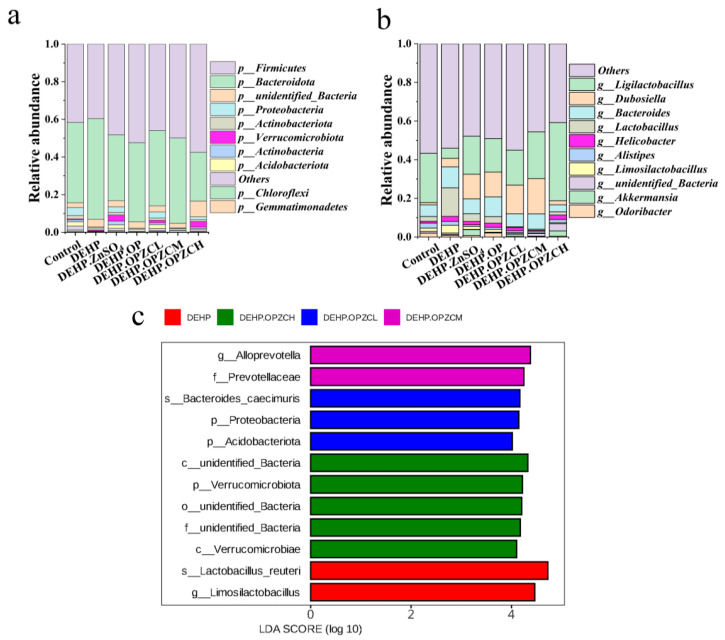
(**a**) Relative abundance of fecal microbes at the phylum level in the seven groups; (**b**) Relative abundance of fecal microbes at the genus level in the seven groups; and (**c**) LEfse analysis of fecal microbiome data.

To further clarify the mechanisms of the role of OPZC in alleviating the reproductive toxicity of DEHP, we investigated the fecal microorganisms. We collected fecal samples at week 6 to assess the composition of the microbiota. The diversity of mice fecal microbiota was measured based on ACE (Figure 9a), Chao1 (Figure 9b), Shannon (Figure 9c), and the Simpson index (Figure 9d). As can be seen from the results, the mice fecal microbiota (ACE, Chao1, and Shannon) richness index of the DEHP and control groups showed significant differences (*p* < 0.05), whereas the addition of ZnSO_4_ and OPZCL significantly altered the overall diversity of the fecal microbiota of the DEHP group (*p* < 0.05). There was no significant change in the OP group and OPZCM group. To determine the varying degree of gut microbiota between different groups, the structural changes in the fecal microbiota were further analyzed using NMDS. NMDS results (Figure 9e) indicated that the DHEP treatment altered gut microbial diversity, while ZnSO_4_ and OPZCL treatments had a positive effect. Figure 10 shows the relative abundance of the top first 10 phylum and 10 genus of the seven groups, respectively. To further identify the biomarkers of differences, we performed a LEfSe analysis.

As shown in Figure 10a, the phylum level analysis indicated that the relative abundance of *Bacteroidota* in the DEHP group was significantly higher than that in the control group (*p* < 0.05), and the difference in the relative abundance of *Firmicutes* was not significant. The supplementation of ZnSO_4_ and OPZCH significantly reduced the relative abundance of *Bacteroidota* (*p* < 0.05). Moreover, we also found that the relative abundance of *Acidobacteriota, Chloroflexi*, *and Proteobacteria* in the DEHP group was significantly lower than that in the control (*p* < 0.05) group. Finally, the OPZCL (*p* < 0.05) group could significantly increase the relative abundance of these three bacteria, and the ZnSO_4_ group was also more effective.

When comparing the DEHP-treated to control mice, at genus level, a significant increase in abundance of *Lactobacillus* and *Bacteroides* were observed (*p* < 0.05) (Figure 10b), which was mitigated by ZnSO_4_, OPZCL, and OPZCH (*p* < 0.05). We also found the relative abundance of *Ligilactobacillus* decreased after DEHP exposure (*p* < 0.05), and OPZCH changed this state. These results suggest that ZnSO_4_ and OPZC treatments improved the intestinal flora community in DEHP-induced reproductive toxicity in mice. Furthermore, the differences in microbiota among the DEHP, OPZCL, OPZCM, and OPZCH groups were compared by LEfSe analysis and LDA scores (Figure 10c). The data showed that *Lactobacillus-reuteri* and *Limosilactobacillus* were dominant in the DEHP group. In additon, the characteristic microorganisms in the OPZCL group were *Bacteroides-caecimuris*, *Acidobacteriota,* and *Proteobacteria*. *Alloprevotella* and *Prevotellaceae* are typical biomarkers of the OPZCM group, while *unidentified Bacteria* and *Verrucomicrobiota* are dominant in OPZCH after DEHP gavage treatment.

**Figure 11:**
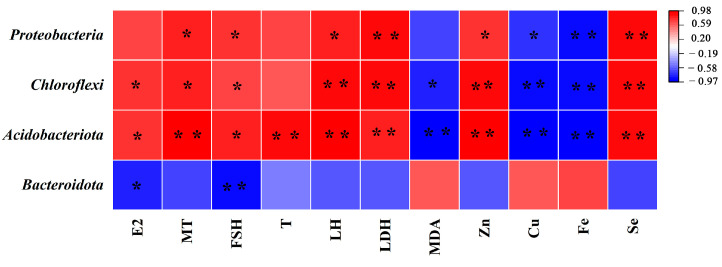
Correlation of gut microbiota and bio-indexes of mice treated with DEHP and OPZCL. Red/Blue indicates that altered indexes are positively/negatively correlated with perturbed gut microbiota. (*) *p* < 0.05, (**) *p* < 0.01.

In addition, Spearman’s correlation analysis was used to find associations between gut microbiota and the bio-indexes of mice treated with DEHP and OPZCL at the phylum level. As shown in the heatmap (Figure 11), *Acidobacteriota*, *Chloroflexi*, and *Proteobacteria* were positively correlated with the index of mitigating reproductive toxicity caused by DEHP, whereas *Bacteroidota* was negatively correlated with the E2, MT, FSH, T, LH, LDH, Zn, and Se index. In general, these results indicate that gut microbiota was significantly correlated with DEHP-induced testicular injury.

## 4. Discussion

Available data indicate that the main male reproductive target organs damaged by DEHP are the testes and epididymis, impairing spermatogenesis by inducing oxidative stress and apoptosis in germ cells [4,34]. It can also alter the release of hypothalamic, pituitary and peripheral hormones, leading to androgenic toxicity [2]. In the present experiment, OPZCL was found to have good therapeutic and mitigating effects on DEHP-induced reproductive injury in adolescent male mice by modulating gut microbiota and decreasing oxidative stress.

It has been found that neonatal exposure to DEHP leads to significant changes in testicular histopathology and an increase in testicular apoptotic cells [35]. In our experiment, DEHP was found to cause a decrease in testicular index, seminal vesicle gland index, anogenital distance in mice, and altered testicle histomorphology, while ZnSO_4_ and OPZCL ameliorated the toxicity of DEHP and could protect against its adverse change. DEHP has a greater effect on liver index, and OPZCL treatment restored the liver index. Similarly, other studies had also found that the exposure of C57BL/6J mice to DEHP resulted in a higher liver index [36]. Furthermore, histopathological analyses showed that DEHP caused a large reduction in spermatogenic cells, abnormal morphology, which was consistent with the findings of previous research [37,38]. ZnSO_4_ and OPZCL restored the morphology of spermatozoa and improved the reproductive capacity of ICR male mice.

Oxidative stress due to toxic substances is considered to be closely related to male infertility [39]. Enzyme activity and spermatogenesis in testicular tissue are closely related and can be used as an indicator for the evaluation of reproductive impairment. In accordance with previous studies [40,41,42], DEHP caused a decrease in CuZn-SOD enzyme activity, T-SOD, LDH, serum zinc ion, and serum MT levels, while increased in MDA levels in ICR mice. Furthermore, we also found that the supplementation with OPZCL restored a healthy reproductive state in the mice. In order to evaluate the protective effects of OPZCL on DEHP-induced testicular injury, reproductive hormones responses in serum were also examined. It has been found that subchronic exposure to low doses of phthalate mixtures impaired reproductive function in male rats, and zinc supplementation inhibited the reduction in serum T, FSH, and LH levels, and ameliorated the structural damage to the testes [43]. In the present experiments, OPZCL significantly restored the levels of FSH, T, LH, and E2 which played a protective effect on the reproductive system of mice.

Trace elements play a key role in maintaining cellular integrity and are particularly important in preventing toxicity. DEHP caused a decrease in zinc and selenium content and an increase in the Cu and Fe content in the testis. Numerous studies have demonstrated that DEHP could cause a decrease in testicular zinc levels, which ultimately leads to testicular atrophy [13,44,45]. CuZn-SOD and T-SOD are often used to reflect the extent of antioxidant damage in the testis. Researchers found that zinc supplementation decreased low-dose Pb-induced lipid peroxidation and increased glutathione, CuZn-SOD levels [46,47]. Studies have shown that the dietary selenium deficiency can reduce sperm motility in rats and lead to sperm abnormalities [48]. Copper exposure resulted in increased MDA concentrations in the testis, mediating oxidative stress that promoted apoptosis and autophagy in testicular cells [49]. Previous studies found that exposure to DEHP before puberty in mice can lead to an increase in iron content in the testes, leading to iron death and ultimately damaging testicular cells [50]. In addition, we found that the supplementation with OPZCL can alleviate the aforementioned metal element homeostasis to a certain extent.

It was found that DEHP affected the levels of relevant metal elements in mice, which also led to the disruption of the intestinal gut microbiota in mice. And it has been recognized as a key causative agent of reproductive disorders, so understanding the effects of DEHP on gut microbes is essential for better management of public health [7,51]. In this study, *Bacteroidota* was significantly elevated, the F/B ratio was also reduced, but not significantly. Additionally, previous studies had found that the DEHP-treated group had higher levels of *Bacteroidota*, with a lower ratio of *Firmicutes* to *Bacteroidota* at the phylum level [52]. Furthermore, similar results were obtained in the present study [36], with *Proteobacteria* and *Actinobacteria* phylum significantly decreasing after DEHP exposure. However, OPZCL prevented DEHP-induced *Bacteroida* increases and *Acidobacteriota*, *Chloroflexi*, and *Proteobacteria* decreases, as well as altered the α-diversity and β-diversity of the intestinal flora. At the genus level, the DEHP exposure increased the relative abundance of *Lactobacillus* and *Bacteroides* and decreased the abundance of *Ligilactobacillus*. A previous study reported that exposure to phthalate during gestation causes severe damage to the reproductive system of male offspring, characterized by an increase in the relative abundance of *Bacteroides* [53,54]. However, OPZCL ultimately improved the intestinal homeostasis. It has been found that exposure of mice to DEHP or BPA (plasticizer) increased the abundance of *Lactobacillus*, which reduces intestinal absorption by promoting the excretion of DEHP and BPA [55,56,57]. Therefore, it was hypothesized that the increase in *Lactobacillus* after DEHP exposure was due to an increase in DEHP-degrading bacteria. In this experiment, the supplementation of OPZCL restored the intestinal flora disordered state by restoring the *Lactobacillus* content similar to that of the control group. Noticeably, *Ligilactobacillus* was increased after OPZC treatment, and has been widely used as a probiotic supplement, either as a growth promoter in animal feed or in human food to improve human health in recent years [58]. Overall, OPZCL had the best therapeutic effect in restoring the gut microbiota disorder induced by DEHP, while OPZCM and OPZCH were less effective than OPZCL.

In conclusion, the OPZC prepared in this paper has a molecular weight range of 600–2000 Da and contains more amino acid chelation sites. The stable chelates were successfully obtained and the molecular docking analysis indicates that glutamine (Gln), glutamate (Glu), histidine (His), and aspartate (Asp) may play an important role in the chelating ability of zinc. OPZC exerted a protective effect against DEHP-induced reproductive injury in mice, and restored the testicular index. Furthermore, OPZC also altered the α-diversity and β-diversity of the intestinal flora as well as the composition of the microbial community, ultimately improving the microbiota metabolism and zinc homeostasis. Among them, low-dose chelating peptide–zinc (OPZCL) has the best effect. The development of OPZCL-rich functional foods for DEHP reproductive injury seems to be a promising alternative treatment.

## Figures and Tables

**Table 1:** Amino acid composition of the OP and the OPZC.

Amino Acids	OP (%)	OPZC (%)
Asp	4.76 ± 0.07	5.28 ± 0.06 **
Thr	2.16 ± 0.04	1.55 ± 0.02
Ser	2.14 ± 0.03	1.86 ± 0.02
Glu	6.83 ± 0.11	6.25 ± 0.08
Gly	2.46 ± 0.05	2.01 ± 0.00
Ala	2.46 ± 0.07	1.55 ± 0.05
Cys	0.24 ± 0.10	0.34 ± 0.09 **
Val	2.21 ± 0.04	1.00 ± 0.00
Met	0.98 ± 0.07	0.38 ± 0.00
Ile	1.99 ± 0.04	0.69 ± 0.02
Leu	2.75 ± 0.04	0.64 ± 0.02
Tyr	1.30 ± 0.02	0.55 ± 0.01
Phe	1.21 ± 0.02	0.39 ± 0.00
His	1.28 ± 0.02	1.42 ± 0.03 **
Lys	3.63 ± 0.07	4.75 ± 0.05 **
Arg	2.59 ± 0.02	3.58 ± 0.04 **
Pro	2.36 ± 0.06	1.31 ± 0.04

Note: ** *p* < 0.05, compared to the OP determined by Student’s *t* test.

**Table 2:** The sequence description of OPZC peptides.

Number	Peptide Sequence	Molecular Weight (Da)	Peptides Score	Potential Active Biopeptide Score
1	GEDGAEGPTGPVGPL	1351.6255	67.25	0.567872
2	KEGLELPEDEEEK	1543.7252	66.11	0.0624502
3	KEGLELPEDEEE	1415.6304	65.16	0.0473078
4	GEPGPEGPAGPIGPR	1386.6891	64.65	0.822365
5	GETGDRGPFGN	1105.4788	61.78	0.509871
6	GLIDEDIEPPR	1252.6299	59.24	0.281908
7	GIVLDSGDGVSH	1154.5568	58.64	0.190025
8	GIVLDSGDGVTH	1168.5724	58.07	0.181258
9	LDVPDEPVHEPTPV	1542.7566	56.92	0.197089
10	GHPGLPGDAGPEGPR	1412.6796	56.23	0.729975
11	GPQGDDGAIGPT	1083.4833	54.45	0.535189
12	LDIERPTYT	1106.5608	54.24	0.125358
13	GPQGHPGLPGDAGPEGPR	1694.8124	54.2	0.815328
14	SETGAGKHVPR	1137.589	54.19	0.171056
15	SHEGYPFPPVSTD	1431.6306	54.18	0.312428
16	DMEGKPSPPGPS	1197.5334	53.75	0.342438

## Data Availability

Data is contained within the article.
